# Use of organotypic or three-dimensional systems with basic fibroblast growth factor for *in vitro* culture of immature collared peccary testicle

**DOI:** 10.1530/RAF-25-0026

**Published:** 2025-08-08

**Authors:** Ana Glória Pereira, Andréia Maria da Silva, Luana Grasiele Pereira Bezerra, Joana Letícia Cottin de Albuquerque, Gabriel Santos Costa Bezerra, Alexsandra Fernandes Pereira, Carlos Eduardo Bezerra de Moura, Pierre Comizzoli, Alexandre Rodrigues Silva

**Affiliations:** ^1^ Laboratory of Animal Germplasm Conservation, Federal Rural University of Semi‐Arid, UFERSA, Mossoro, Brazil; ^2^ Laboratory of Animal Biotechnology, UFERSA, Mossoro, Brazil; ^3^ Department of Animal Sciences, UFERSA, Mossoro, Brazil; ^4^ Smithsonian’s National Zoo and Conservation Biology Institute, Washington, District of Columbia, USA

**Keywords:** Biobank, gonadal tissues, collared peccary, wildlife

## Abstract

**Abstract:**

The objective was to evaluate the effects of different culture systems and the addition of fibroblast growth factor (bFGF) during *in vitro* culture (IVC) of testicular tissue fragments from prepubertal collared peccaries. Testes from five individuals were collected, dissected, and cultured for up to 56 days (34°C and 5% CO_2_) in Dulbecco’s Modified Eagle’s Medium (DMEM), supplemented or not with FGF at 10 ng/mL, in organotypic (ORG) or 3D system culture. Samples were evaluated every 14 days for histomorphology, cell viability, DNA integrity, and proliferative activity. Overall, the ORG system without FGF addition was the best to preserve testicular fragment histomorphology, viability, and DNA integrity during IVC. However, the 3D system, regardless of the presence of FGF, impaired the DNA integrity of testicular cells in all culture periods analyzed. Regarding cell proliferation, at 14 days the ORG group without addition of FGF showed a percentage of Ki-67 positive cells indicative of proliferation similar to the non-cultured group, while the other treatments reduced proliferation. However, at 28 days a reduction in proliferation was observed in this same group and an increase in proliferation in the others. Cell proliferation was reduced in all groups at 42 days (*P* < 0.05). In summary, we suggest the use of the organotypic system for long-term culture of testicular fragments of prepubertal collared peccaries. In addition, FGF supplementation to the culture medium does not seem to be essential.

**Lay summary:**

Animals do not produce sperm cells before puberty. However, in case of unexpected death of young individuals carrying critical genes for the diversity and sustainability of an animal population, sperm cells can be obtained by recovering and culturing tissue from the testes in proper laboratory conditions. Resulting sperm cells can then be used to produce embryos using IVF methods. The goal of the present work was to find the best culture conditions to keep pieces of testicular tissue alive for extended periods of time using the collared peccary as a model. Two different methods were tested. The first approach was to place a piece of tissue on top of a gel that was rich in nutrients, similar to the natural supply to the tissue. This method is called organotypic culture. The second approach was to recreate a more natural environment by embedding the tissue inside the gel, which is known as 3D culture. Overall, the organotypic culture was the best way to keep the tissues alive for 56 days. This is a major step forward to allow the production of sperm cells from peccaries in the laboratory.

## Introduction

The world has been experiencing numerous climate changes that have had a huge impact on its ecosystems. In addition, human action, indiscriminately, has negatively impacted the survival of various wild animal species in different biomes (Comizzoli & Holt 2024). One of the greatest examples of this action has been the collared peccary (*Pecari tajacu* Linnaeus, 1758), whose populations are in sharp decline in Mesoamerica (Thornton *et al.* 2020), although they are still globally classified as least concern (Gongora *et al.* 2011). Given the ecological importance of peccaries as members of large carnivore food chains, as well as their role as seed dispersers and soil aerators (Desbiez *et al.* 2012), the development of strategies for their conservation has been a constant concern for numerous researchers.

Among conservation strategies, collared peccaries are particularly noteworthy for serving as a model for the widespread development of innovative assisted reproduction techniques such as cryopreservation (Silva *et al.* 2019, 2021) and culture (Silva *et al.* 2024) of testicular tissues, which can be extrapolated to countless other wild species. Testicular technology opens up numerous perspectives for wildlife conservation since it would allow the recovery, storage, and cultivation of spermatogonia, providing an inexhaustible source of male gametes to be used in other biotechnologies (Peris-Frau *et al.* 2023). Despite advances in the conservation of peccary testicular germplasm (Silva *et al.* 2019, 2021), providing the necessary conditions for the reestablishment of testicular function and consequent progression of spermatogenesis is still a challenge. Furthermore, low testicular survival under ex situ conditions for a period long enough to mimic physiological spermatogenesis, especially in wild animals (Sharma *et al.* 2022*a*,*b*), limits the development of techniques applied to *in vitro* sperm production.

The establishment of an *in vitro* culture (IVC) system must be capable of maintaining viability and promoting the proliferation of testicular cells, which would allow the use of cryopreserved or fresh samples from individuals who died suddenly (Ibtisham *et al.* 2023). The first step toward the establishment of testicular IVC in a wild species was conducted by Silva *et al.* (2024), who demonstrated the efficiency of different media supplemented with 10 ng/mL of glial cell-derived neurotrophic factor (GDNF) during 28-day culture in an organotypic system, supporting cell survival and proliferation in testicular fragments from immature peccaries. Despite its promising results, the use of the organotypic system with an agarose gel-based extracellular matrix enables cell detachment from the seminiferous tubules, as observed in pigs (Ibtisham *et al.* 2023) and mice (Sato *et al.* 2015). A 40-day 3D culture in which the testicular fragment was encapsulated was recently shown to overcome this challenge by enabling sperm production from seminiferous tubules of immature mice, thus reducing cell loss (Celiz *et al.* 2023). However, there are no reports of spermatogenesis in 3D IVC of testicular fragments in domestic or wild animals.

IVC allows precise environmental control, preserving testicular microstructure and enabling the study of isolated molecular factors affecting spermatogenesis (Sato *et al.* 2012, Ibtisham & Honaramooz 2020). In peccaries, the GDNF supplementation is highlighted as essential for testicular IVC (Silva *et al.* 2024). This is a factor released by Sertoli cells that helps regulate the proliferation and differentiation of spermatogonia (Hofmann *et al.* 2005). Other growth factors are also interesting alternatives to be added to the testicular IVC systems, such as basic fibroblast growth factor (bFGF), which regulates GDNF production in Sertoli cells (Simon *et al.* 2007). In fact, the combination of GDNF and bFGF provided the maintenance of testicular integrity and gonocyte number and induced *in vitro* spermatogenesis in testicular fragments from prepubertal pigs (Ibtisham *et al.* 2023), the domestic species most closely related to the peccaries (Theimer & Keim 1998).

The aim of this study was to evaluate the efficiency of a three-dimensional IVC system in comparison with the organotypic cultures of testicular tissues from immature individuals (as a basis for future experiments of *in vitro* spermatogenesis). Furthermore, the aim was to verify the effect of bFGF supplementation to the medium during a long period of IVC on the histomorphology, viability, DNA integrity, and cell proliferation in the testes of prepubertal collared peccaries.

## Materials and methods

### Animal ethics and husbandry

All experiments were conducted following the Animal Ethics Committee of the Federal Rural University of Semi-Arid – UFERSA (no. 24/2023) and Chico Mendes Institute for Biodiversity Conservation (no. 72170). All males belonged to the Wild Animal Multiplication Center (CEMAS – UFERSA, Mossoró, RN, Brazil), which is registered at the Brazilian Institute of Environment and Renewable Natural Resources (IBAMA) as a scientific breeding center (no. 1478912) and located at 5°10′ S and 37°10′ W at an altitude of 16 m, characterized by a semiarid tropical climate.

### Testis collection and experimental design

**Figure 1:**
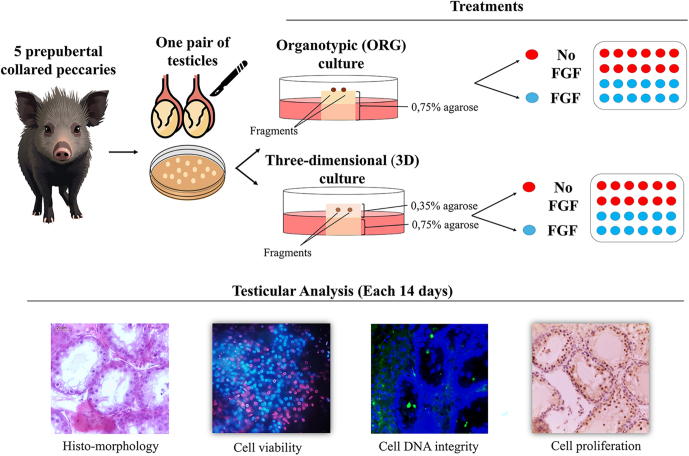
Experimental design to evaluate different IVC systems of testicular fragments from prepubertal collared peccaries. Testicular tissue fragments from prepubertal males were collected, prepared, and cultured under different conditions, including fresh controls, and organotypic or three-dimensional culture systems with or without growth factor supplementation.

Gonads from five prepubertal males (3–6 months of age) were collected. Pairs of testes were washed in saline (0.9% NaCl) and transported in a solution consisting of Dulbecco’s Modified Eagle’s Medium (DMEM) and a 1% antibiotic–antimycotic combination to the laboratory at 22°C. Each of the five animals used was considered a single replicate and was collected at different times. Testes were dissected from the surrounding tissues and washed with three successive baths of the transport solution. Then, testes were cut into small pieces of 1 × 1 × 1 mm^3^. A total of 264 fragments per pair of testis were randomly divided among the five treatment groups: fresh non-cultured IVC, organotypic (ORG) with or without FGF, and three-dimensional (3D) with or without FGF addition (Fig. 1). All treatments were distributed in 24-well cell culture plates (four per pair of testes) and cultivated in a humid atmosphere with 5% CO_2_ at 34°C for a total of 56 days. This IVC time was established according to the physiological spermatogenesis of collared peccaries, which lasts 55 days on average (Costa *et al.* 2010). Fragments from all groups were evaluated every 14 days (Fig. 1).

### *In vitro* culture of testicular fragments in different systems

The protocol for the ORG IVC system used was described by Silva *et al.* (2024). We dissolved agarose powder (A013, Sigma-Aldrich, USA) in sterilized distilled water at 0.75% and poured it into 10 cm diameter petri dishes, which were kept at room temperature until the gel solidified. 1 × 1 × 1 cm^3^ cubes were made and placed inside the wells of the culture plates, one cube per well. Then, 500 μL of the medium per well was placed up to 12 h before the start of the culture.

For the three-dimensional culture system, the testicular fragments were allocated between two agarose gel platforms. The first platform was made as established for the organotypic system, at a concentration of 0.75% w/v agarose. The second platform was prepared at the time of gonad collection; the 0.75% agarose solution was diluted in culture medium to a final concentration of 0.35% w/v agarose and placed on the first platform. Subsequently, all testicular fragments were immersed in this solution until solidification so that they were surrounded by a 3D configuration (Celiz *et al.* 2023). Agarose from both the organotypic and 3D systems was sectioned into 1 × 1 × 1 cm^3^ cubes and placed in 24-well plates containing 500 μL of culture medium (Fig. 1).

Fragments (two per well) were cultured in 24-well plates for a total of 56 days at 34°C in a humidified atmosphere of 5% CO_2_. Each well was filled with 500 μL of culture medium, consisting of DMEM (11885084, Gibco, USA) supplemented with 10% fetal bovine serum (FBS, 12657029, Thermo Fisher, USA), 25 mM HEPES (Sigma, H6147), 2 μL/mL insulin–transferrin–selenium (ITS; Gibco, 41400-045), 1% NEAA solution (Sigma, M7145), 1% vitamin solution (Gibco, 11120), 0.2% bovine serum albumin (Sigma-Aldrich, A7906), 1% antibiotic–antimycotic solution (Gibco, 15240), 10 ng/mL LH/FSH (Pluset®, Hertape Calier, Brazil), 20 ng/mL mEGF (Sigma, E4127), and 10 ng/mL GDNF (512-GF, Thermo-Fisher, USA), with or without 10 ng/mL bFGF (100-18B, Thermo-Fisher, USA). A complete medium change was performed every 72 h, totaling four changes for 14 days. Every 14 days, fragments were removed for analysis.

### Testicular fragment histomorphology

Fresh uncultured and cultured testicular fragments were fixed in Bouin’s solution for 12 h, embedded in paraffin blocks, sectioned (5.0 μm thickness), stained with hematoxylin-eosin, and evaluated under a light microscope at 400×, (CX 31 RBSFA, Olympus, Japan). In the morphological parameters, each seminiferous tubule was analyzed individually for tubular structure, ruptures of the basement membrane, swelling, tubular cell loss, separation of the basal membrane, and vacuolation, which were assessed according to a score scale established (Table 1) (Silva *et al.* 2024). For each parameter, five seminiferous tubules were evaluated in six different fields, which corresponded to a total of 30 seminiferous tubules for each treatment (Silva *et al.* 2019). Testicular tissue fragments with a total score of 3 were considered morphologically normal. Fragments with a total score of 1 were considered degraded/degenerated.

**Table 1:** Histomorphological parameters of seminiferous tubules in testicular fragments from collared peccaries*.

Parameter	Scores		
	3	2	1
Shrinkage from basal membrane	No shrinkage	Partly shrinkage (<50%)	Mostly shrinkage (>50%)
Tubular cell swelling	No swelling	>50% cells without swelling	>50% cells with swelling
Rupture from the basal membrane	No rupture	Partly ruptured (<50%)	Mostly ruptured (>50%)
Tubular cell loss	No cell loss	<75% cell type loss	>75% cell type loss
Tubular structure	Structure intact	All cell types of presents although slightly disordered structure	Random distribution of remaining cells

* Silva *et al.* (2024).

### Cell viability in testicular fragments

Testicular cells were isolated from the fragments by enzymatic digestion (Abrishami *et al.* 2010). Briefly, uncultured fragments were incubated with 0.2% collagenase type IV at 37°C for 10 min, followed by the addition of an equal volume of DMEM plus FBS to stop the enzyme action, and centrifuged at 114 *g* for 5 min. For cultured fragments, enzymatic digestion was dispensed with due to disaggregation of the fragments. Volumes of 10 μL of cell pellets or fragments were incubated in a mixture of 3 μL propidium iodide (0.5 mg/mL in phosphate-buffered saline, PBS) and 5 μL Hoechst 33342 (40 μg/mL in PBS). The final volume was 18 μL, resulting in a final concentration of 0.33 mg/mL for propidium iodide and 11.11 μg/mL for Hoechst 33342. The sample was incubated for 10 min at 37°C to detect nonviable testicular cells (red staining) and count total cells (blue nuclear staining), respectively. Samples were evaluated under an epifluorescence microscope (×400; Leica, Sweden) by counting a total of 100 cells and sorted into each treatment group.

### Cell DNA integrity in testicular fragments

Testicular fragments were fixed overnight in 4% paraformaldehyde solution, embedded in paraffin, serially sectioned (5 μm thickness), mounted on slides, and evaluated using the terminal deoxynucleotidyl transferase biotin-dUTP nick-end labeling kit (TUNEL Assay Kit 11684809910, Roche, Switzerland) as proposed by Silva *et al.* (2021). The TUNEL reaction mixture was prepared using the enzymatic solution composed of 5 μL of terminal deoxynucleotidyl transferase (TdT) and 45 μL of labeling solution composed of nucleotide polymers. The sections were incubated with 50 μL of the TUNEL reaction mixture for 1 h at 37°C inside a humidified and dark container. A negative control, in which only 50 μL of labeling solution without terminal transferase was used instead of the TUNEL reaction mixture, was included. The positive control was performed by incubating the cells with 5 μL of recombinant DNase I (Sigma-Aldrich) and 45 μL of 0.05% Triton X-100 in PBS for 10 min before the labeling procedures to induce DNA breaks. The nuclei of all cells were stained with Hoechst 33342 (1:100, Sigma-Aldrich) for counterstaining. We evaluated ten images per experimental group, which were captured using an epifluorescence microscope. The images were evaluated by ImageJ software, and the cells from ten images/treatment were counted.

A minimum of 1,000 cells were analyzed per sample to determine the level of nuclear DNA fragmentation damage. TUNEL-positive cells showed green fluorescence and were classified as damaged, while TUNEL-negative cells, counterstained with blue fluorescence, were classified as normal.

### Cell proliferation in testicular fragments

To evaluate the proliferative capacity of testicular cells, an immunohistochemical assay for detection of Ki-67 protein was used (Zhao *et al.* 2018). Paraffin-embedded samples were sectioned at 5 μm, dewaxed, and rehydrated in xylene and distilled water. Then, antigen retrieval was conducted in a microwave oven. Endogenous peroxidase activity in collagens was quenched by 3% hydrogen peroxide at room temperature for 30 min, and the collagens were then blocked with blocking solution (SC-516214, UltraCruz^®^, Brazil) in a humidified chamber for 30 min at room temperature. The samples were subsequently incubated with Ki-67 primary antibody (PA5-19462, Invitrogen^®^, USA) at a dilution of 1:300 overnight at 4°C in a humidified chamber. They were then washed with PBS and incubated with secondary antibody (Invitrogen^®^, 616520) at a dilution of 1:250 for 1 h in a dark, humidified chamber at room temperature. They were then stained by the addition of 3,3′-diaminobenzidine and counterstained with hematoxylin.

To quantify Ki-67-positive cells, histological sections were evaluated using a computerized image analysis system with ImageJ software (National Institutes of Health, USA). Each nucleus immunostained with brown Ki-67 antibody was quantified. Then, the purplish-blue nuclei stained with hematoxylin were also counted. The percentage of the area stained with Ki-67 was verified.

### Statistical analysis

Each of the five animals used was considered a single replicate. Within and between replicates, all results were expressed as mean ± standard error. For all analyses, the Jamovi statistical software (SAS Institute Inc., USA) was used, considering a significance value of *P* < 0.05. Data verification for normality was performed using the Kolmogorov-Smirnov test, and for homoscedasticity, using the Levene test. The existence of variation between morphology, DNA integrity, and cell proliferative potential was verified using the Kruskal–Wallis test. We verified the effect of treatment on the viability of testicular fragments by analysis of variance followed by two-way ANOVA with repeated measures over time, and tests between treatments were performed using the Tukey test.

During periodic medium changes, fungal contamination was detected in some samples. Contaminated wells were isolated to avoid excessive handling and reduce the source of contamination for other samples, which affected the results on day 56. Therefore, contaminated samples were excluded from the experiment, which did not allow us to perform statistical analyses including the samples from that assessment at 56 days.

## Results

### Testicular fragment histomorphology

**Figure 2:**
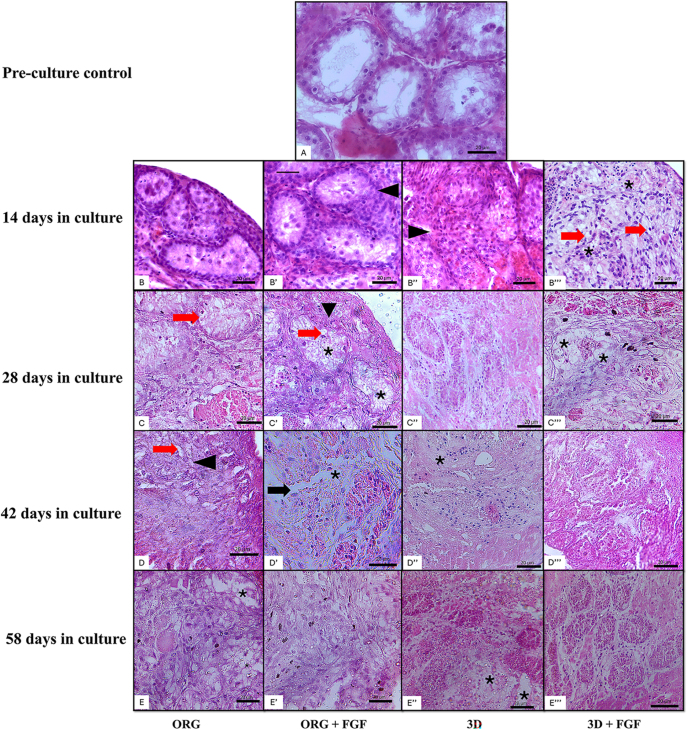
Histomorphological evaluation of testicular tissues of prepubertal peccaries (*n* = 5) cultured by organotypic (ORG) and three-dimensional (3D) systems. (A) Control group, not cultured, and (B–E^‴^) groups cultured for 14 (B–B^‴^), 28 (C–C^‴^), 42 (D–D^‴^), and 56 days (E–E^‴^) using the ORG (B, C, D, E), ORG with FGF (B′, C′, D′, E′), 3D (B″, C″, D″, E″), and 3D with FGF (B^‴^, C^‴^, D^‴^, E^‴^) culture systems. Black arrows show basement membrane shrinkage. Arrowheads show basement membrane rupture. Red arrows indicate cell swelling. Asterisks show loss of tubular cells. Scale bar: 20 μm.

**Figure 3:**
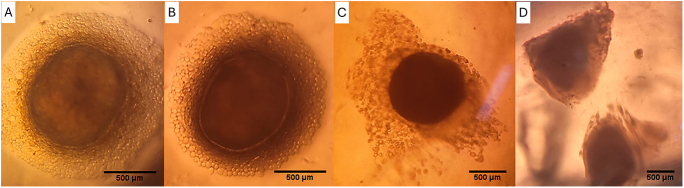
Photomicrographs of testicular fragments of prepubertal collared peccaries cultured *in vitro* for 56 days in different culture systems: (A) organotypic (ORG), (B) ORG with FGF, (C) three-dimensional (3D), and (D) 3D with FGF. Testicular cells can be observed around the fragments and aggregated into the agarose gel matrix. Scale bar: 500 μm.

Morphology results are shown in Table 2 and Fig. 2. The ORG system supplemented with FGF provided the lowest incidence of basement membrane separation at 14 days (*P* < 0.05), but it was similar to the uncultured control (*P* > 0.05). At 28 days, ORG and 3D with FGF were more efficient in preventing basement membrane shrinkage and tubular cell loss when compared to culture without FGF (*P* < 0.05). At 42 days, the group cultured in the 3D system prevented tubular cell swelling in comparison to the ORG system (*P* < 0.05). At 42 days, the ORG and 3D groups cultured without FGF showed better preservation of membrane integrity, cell loss, and seminiferous tubule structure. However, the addition of FGF to the medium impaired the tubular structure and increased the proportion of lost cells, tubular vacuolization, and membrane rupture, regardless of the culture system used. An increase in cell loss was observed proportional to the progression of the culture, and it was possible to visualize the absence of intratubular cells, which was greater at 56 days (Fig. 2), impairing the evaluation of other morphological parameters. This cell loss was due to the migration of intratubular cells to the agarose gel matrix, as observed in Fig. 3.

**Table 2:** Mean (±SEM) values for morphological evaluation scores (from 3 – normal – to 1 – degenerated) on immature collared peccary testicular tissues (*n* = 5) cultured *in vitro* for 56 days using organotypic (ORG) or three-dimensional (3D) systems with medium supplemented with different FGF (0 or 10 ng/mL) concentrations.

Culture day/system	FGF (ng/mL)	Shrinkage from BM	TC swelling	Rupture from the BM	TC loss	Tubular structure
0						
Controls^†^		2.71 ± 0.03^A^	2.51 ± 0.04^A^	2.80 ± 0.03^A^	2.92 ± 0.01^A^	2.60 ± 0.03^A^
14						
ORG						
	0	2.34 ± 0.03^Bb^	2.06 ± 0.03^Ba^	2.49 ± 0.04^BCa^	2.50 ± 0.03^Ba^	2.20 ± 0.03^Ba^
	10	2.68 ± 0.03^Aa^	2.21 ± 0.04^Ba^	2.62 ± 0.03^Ca^	2.45 ± 0.04^Ba^	2.14 ± 0.04^Ba^
3D						
	0	2.30 ± 0.05^BDb^	1.79 ± 0.05^Cb^	2.29 ± 0.05^Db^	2.03 ± 0.05^Cb^	1.65 ± 0.04^Db^
	10	1.58 ± 0.07^Cc^	1.42 ± 0.06^Dc^	1.91 ± 0.08^Ec^	1.63 ± 0.07^Dec^	1.38 ± 0.05^CDc^
28						
ORG						
	0	1.95 ± 0.07^CFab^	1.55 ± 0.05^Cdab^	1.94 ± 0.06^EFbc^	1.68 ± 0.05^Dea^	1.62 ± 0.06^CDab^
	10	2.11 ± 0.06^BFa^	1.78 ± 0.06^Cac^	1.96 ± 0.06^Eb^	1.74 ± 0.05^Da^	1.72 ± 0.05^Cda^
3D						
	0	1.61 ± 0.07^Cb^	1.41 ± 0.05^Db^	1.89 ± 0.08^EFc^	1.40 ± 0.06^Eb^	1.41 ± 0.06^Db^
	10	2.01 ± 0.07^DFa^	1.75 ± 0.05^Cc^	2.24 ± 0.06^DFGa^	1.61 ± 0.05^Ea^	1.52 ± 0.04^Dab^
42						
ORG						
	0	2.26 ± 0.07^BFa^	1.63 ± 0.06^CDb^	2.34 ± 0.07^BDa^	1.91 ± 0.06^CDa^	1.83 ± 0.06^Cab^
	10	1.95 ± 0.05^Fb^	1.54 ± 0.04^CDb^	2.05 ± 0.05^DEb^	1.68 ± 0.06^DEb^	1.64 ± 0.05^CDbc^
3D						
	0	2.13 ± 0.05^BFab^	2.14 ± 0.07^Ba^	2.40 ± 0.05^BCGa^	2.09 ± 0.06^Ca^	2.07 ± 0.07^Ba^
	10	2.15 ± 0.08^BFab^	1.74 ± 0.07^CDb^	2.06 ± 0.06^DEb^	1.50 ± 0.06^Eb^	1.53 ± 0.06^CDc^
56						
ORG						
	0	2.08 ± 0.08*	1.35 ± 0.06*	1.92 ± 0.08*	1.58 ± 0.06*	1.47 ± 0.06*
	10	1.50 ± 0.07*	1.05 ± 0.02*	1.70 ± 0.09*	1.12 ± 0.04*	1.13 ± 0.04*
3D						
	0	1.75 ± 0.09*	1.88 ± 0.04*	1.84 ± 0.05*	1.00 ± 0.00*	1.00 ± 0.00*
	10	1.39 ± 0.10*	1.35 ± 0.10*	1.78 ± 0.08*	1.00 ± 0.00*	1.00 ± 0.00*

^ABCDEFG^ Different uppercase letters indicate differences (*P* < 0.05) within each treatment across culture time points (0, 14, 28, 42, or 56 days of culture). ^abcd^ Different lowercase letters indicate differences (*P* < 0.05) among treatments within the same time point.

* No comparative tests were performed for 56-day evaluated samples due to fungal contamination.

^†^ Non-cultured.

BM, basal membrane; TC, tubular cell.

### Cell viability in testicular fragments

**Figure 4:**
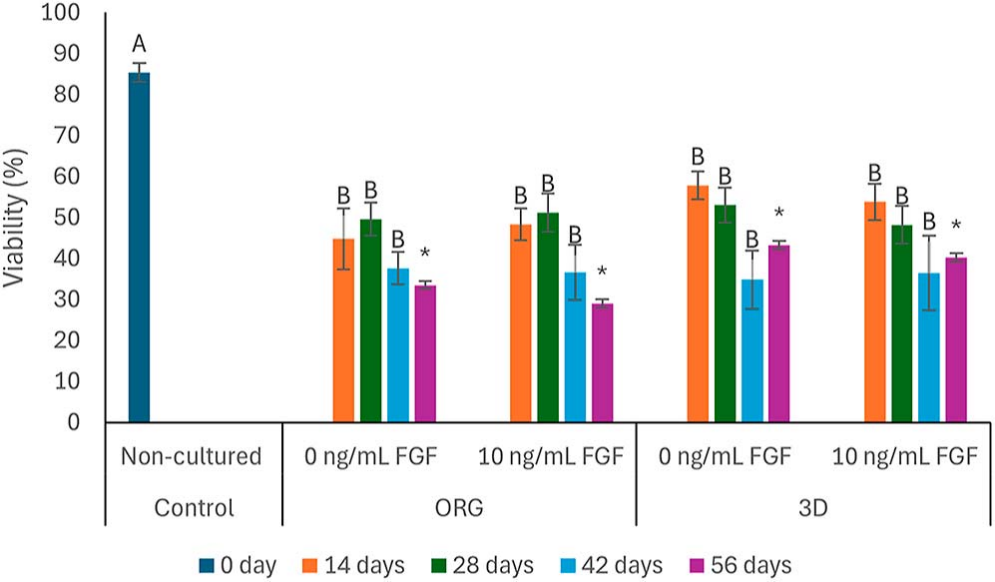
Mean (±SEM) values for germ cell viability of prepubertal collared peccary (*n* = 5) testicles cultured *in vitro* for 56 days in organotypic (ORG) or three-dimensional (3D) systems in media supplemented with different FGF (0 or 10 ng/mL) concentrations. ^AB^ Different uppercase letters above bars indicate differences in the treatments during the culture (0, 14, 28, 42, or 56 days). * Data were not statistically analyzed due to fungal contamination.

A significant reduction (*P* < 0.05) in testicular cell viability was observed when comparing the uncultured control group with those subjected to all culture treatments (Fig. 4). However, during all IVC, the cell viability of the cultured samples was stable (*P* > 0.05), not being influenced by the treatment used, with values ranging from 30 to 60%.

### Cell DNA integrity in testicular fragments

Table 3 shows the mean values (±SEM) for DNA integrity of testicular cells. We observed a decrease in the percentage of cells with DNA damage, which ranged from 0 to 90%. Regardless of the treatment, the cultured groups showed a higher percentage of cells with damaged DNA compared to the uncultured control (*P* < 0.05). However, the ORG group without FGF addition was the only one that maintained DNA integrity and a low percentage of damaged cells (2.2–13.0%) throughout the culture.

**Table 3:** Percentage of cells with damaged DNA in collared peccary testis (*n* = 5) cultured *in vitro* for 56 days in organotypic (ORG) or three-dimensional (3D) systems in media supplemented with different FGF (0 or 10 ng/mL) concentrations.

Culture day/system	FGF (ng/mL)	Mean ± SEM	Minimum	Maximum
0				
Control*	0	2.5 ± 0.4^A^	0.0	15.9
14				
ORG				
	10	7.2 ± 1.0^Ba^	0.0	29.0
	0	26.1 ± 2.4^CEb^	0.0	70.7
3D				
	0	32.7 ± 1.6^Eb^	7.9	60.7
	10	17.11 ± 3.3^BDa^	0.0	90.0
28				
ORG				
	0	2.2 ± 0.3^Aa^	0.0	11.9
	10	17.5 ± 2.1^CFb^	5.6	50.0
3D				
	0	36.3 ± 2.0^Ec^	23.1	56.8
	10	10.7 ± 1.2^BDFb^	0.0	37.5
42				
ORG				
	0	13.0 ± 3.6^BCa^	2.0	70.4
	10	22.7 ± 3.8^CDEb^	6.4	57.5
3D				
	0	41.2 ± 1.9^Ec^	2.1	68.2
	10	20.0 ± 2.6^CDab^	0.0	76.5

^AB^ Different uppercase letters above bars indicate differences in the treatments during the culture days (0, 7, 14, 21, or 28 days); ^ab^ Different lowercase letters above bars indicate differences among treatments on the same day (*P* < 0.05).

* Non-cultured.

### Cell proliferation in testicular fragments

The results (mean ± SEM) regarding cell proliferation are shown in Table 4, with the percentage of Ki-67-positive cells ranging from 0 to 86.7%. At 14 days, a reduction in cell proliferation was observed in all treatments when compared to the uncultured control (*P* < 0.05), except in the group cultured in the ORG system without FGF, which was the only one with a percentage of Ki-67-positive cells similar to the control (*P* > 0.05).

**Table 4:** Percentage of cell proliferation in Ki-67 positive cells of prepubertal collared peccary testis (*n* = 5) cultured *in vitro* for 56 days in organotypic (ORG) or three-dimensional (3D) systems in media supplemented with different FGF (0 or 10 ng/mL) concentrations.

Culture day/system	FGF (ng/mL)	Mean	Minimum	Maximum
0				
Controls*	0	44.0 ± 2.1^A^	10.8	71.9
14				
ORG				
	10	38.3 ± 3.0^Aba^	11.1	75.0
	0	22.1 ± 1.7^Bb^	12.5	38.2
3D				
	0	11.5 ± 1.2^CDc^	4.3	16.3
	10	30.1 ± 3.1^Bcab^	3.4	86.7
28				
ORG				
	0	17.6 ± 1.8^BCb^	7.4	26.7
	10	37.6 ± 5.4^ABCa^	4.1	81.8
3D				
	0	35.3 ± 5.4^ABCab^	4.1	83.3
	10	29.3 ± 5.3^ABCab^	4.7	74.2
42				
ORG				
	0	6.9 ± 2.4^Da^	0.0	40.0
	10	8.4 ± 1.7^Da^	0.0	32.0
3D				
	0	12.9 ± 2.0^BCDa^	9.0	16.0
	10	10.0 ± 2.2^Da^	0.0	52.6

^ABCD^ Different uppercase letters indicate differences (*P* < 0.05) within each treatment across culture time points (0, 14, 28, 42, or 56 days of culture); ^abc^ Different lowercase letters indicate differences (*P* < 0.05) among treatments within the same time point.

* Non-cultured.

However, at 28 days, there was an increase in cell proliferation in the groups cultured by the ORG system with FGF and in those cultured in the 3D system, regardless of the addition of FGF, becoming similar to the uncultured control (*P* > 0.05). The ORG system without FGF reduced the percentage of Ki-67-positive cells in relation to the control (*P* < 0.05). At 42 days, all cultured groups showed a reduction in proliferation indicated by positive staining with Ki-67 and did not differ from each other.

## Discussion

The results of the present study demonstrate that the ORG system is adequate for the IVC of testicular fragments from prepubertal collared peccaries and that FGF supplementation is not essential for *in vitro* testicular development. As previously reported (Guimarães *et al.* 2013), collared peccaries begin puberty around 11 months of age, and their spermatogenesis lasts an average of 55 days (Costa *et al.* 2010). Thus, based on the duration of this important physiological cycle, we cultured the testicular fragments for 56 days. Our team has already studied the ideal conditions for the ORG culture of testicular fragments from prepubertal collared peccaries for a period of 28 days, highlighting the use of DMEM supplemented with 10 ng/mL GDNF as ideal for the proliferation of germ cells (Silva *et al.* 2024). However, the 28-day period was not sufficient to observe the complete adaptation of the testis to IVC. Thus, the present work emerges as a promising tool for the study of spermatogenesis in collared peccaries and the *in vitro* production of germ cells.

We acknowledge that there is considerable biological diversity in reproductive traits both across species and even among individuals from the same species. However, phylogenetic relationships provide a framework for identifying species that are evolutionarily closer to others, allowing for the extrapolation of certain biological insights (Wu *et al.* 2021). While most reproductive studies have traditionally been conducted in domestic animals (Gholami *et al.* 2018, Celiz *et al.* 2023, Ibtisham *et al.* 2023), these models may not always accurately reflect the physiology of wild species due to evolutionary divergence. Therefore, using a species such as the collared peccary, a wild species with well-documented reproductive biology as a model, can provide more relevant data for understanding the reproductive mechanisms of other phylogenetically related wild species, especially other Tayassuids. This approach is particularly important for conservation breeding programs and for advancing our understanding of reproductive diversity in non-model and endangered species.

To our knowledge, this is the first time that the three-dimensional culture system has been used for a long period to culture testicular fragments. Its use is common in the culture of isolated cells (Abu Elhija *et al.* 2012) and seminiferous tubules (Gholami *et al.* 2018). The only report of the use of this system in testicular fragments was only 48 h long (Celiz *et al.* 2023). Thus, our results demonstrate the effects of this system on germ cells and testicular architecture. Celiz *et al.* (2023) observed that samples cultured in ORG showed less nuclear condensation than those cultured in 3D. They reported that the structure of the agarose gel matrix in the 3D system can retain waste produced during culture and induce nutrient and oxygen deficit, causing oxidative stress and nuclear pyknosis. In addition, ORG was better than 3D concerning cell proliferation and percentage of collagen fibers. However, the short cultivation period, only 48 h, was not sufficient to analyze the adaptation of the fragment to the system used, highlighting the need for studies with long cultivations.

In peccaries, ORG and 3D with FGF preserved the integrity of the basement membrane, also preventing the loss of tubular cells for up to 28 days. However, at 42 days, the addition of FGF to the medium apparently impaired morphological integrity and increased cell loss. Silva *et al.* (2024) report that the testis needs to adapt to ex situ conditions and that, in the first weeks of culture, it undergoes morphological changes and can reduce cellular activity. Intense cell migration was also observed, with cells detaching from the seminiferous tubules and adhering to the agarose gel matrix, thus impairing morphological parameters throughout the culture. The same occurred in the first report of IVC of testicular fragments from prepubertal collared peccaries carried out by our team, where we reported the detachment of cells from the seminiferous tubules and their migration to the agarose gel matrix over 28 days of culture (Silva *et al.* 2024). An important step toward obtaining sperm *in vitro* from testicular fragments of prepubertal collared peccaries would be to find a way to recover these detached cells adhered to agarose and analyze their functionality. Studies indicate that testicular cells isolated from cultured testes from domestic pigs tend to regroup and form organoids with a structural organization similar to that of the testis (Sakib *et al.* 2019). We do not know whether the same occurs with the cells that leave the fragment. Therefore, we believe that this is a gap to be filled.

Another important factor that may be linked to the morphological changes suffered by the testes throughout the IVC is the reduction in the number of seminiferous tubules, which has already been observed in prepubertal pigs (Ibtisham *et al.* 2023). In adult sheep, a reduction in the diameter of the seminiferous tubules of the testicular fragments cultured by both ORG and 3D was observed, a fact that was observed even after only 48 h of culture (Celiz *et al.* 2023). However, due to the tubular degeneration observed mainly in the last weeks of culture, it was not possible to analyze the seminiferous tubules in isolation.

We found that the addition of FGF to the culture medium impaired tubular integrity and increased the rate of cell loss, especially in the last weeks of culture. Although this growth factor promoted positive effects on the morphology of testicular fragments of 1-week-old neonatal pigs cultured *in vitro* (Ibtisham *et al.* 2023), these effects were not observed in collared peccaries. FGFs are expressed in a tissue-specific manner and bind to specific receptors (FGFRs) with tyrosine kinase activity, known as FGFR1, FGFR2, FGFR3, and FGFR4 (Gong 2014). In rodents, FGF-2 expression has been detected in both Sertoli cells and germ cells, with FGF-2 secreted by Sertoli cells regulating germ cell metabolism (Gómez *et al.* 2012), while FGF-2 secreted by germ cells plays a regulatory role in Sertoli cell function (Regueira *et al.* 2015). Furthermore, this factor is essential for the differentiation of gonocytes into spermatogonia (Pui & Saga 2017). During spermatogenesis, its absence leads to deregulated sperm production and altered sperm morphology and function (Saucedo *et al.* 2018). Both Sertoli and germ cells likely produce sufficient levels of FGF, making external supplementation unnecessary. In addition, we believe that the age of the animal may influence the response of the testis to FGF, since in mice the action of FGF was evidenced before the onset of spermatogenesis, in the transition from gonocytes to spermatogonia (Pui & Saga 2017). Therefore, studies in neonatal or fetal collared peccaries would be necessary to analyze the role of FGF before gonocyte differentiation. This is the first time that FGF has been used in the testicular IVC of this species, and we obtained important information about its effects on testicular histomorphology.

The viability, or structural integrity, of the plasma membrane is vital for cell survival and maintenance of its functionality, since the membrane is responsible for the selective transport of molecules, and its structure and composition can undergo modifications to provide resistance to injury in different cellular contexts (Ammendolia *et al.* 2021). In our study, there was a reduction in the viability of the cultured group of samples compared to the uncultured group, regardless of the system used. However, the cultured group maintained its viability throughout the culture. Silva *et al.* (2024) report that testicular samples from prepubertal collared peccaries tend to decrease their viability in the first weeks of culture. However, the testis appears to adapt to ex situ conditions over the days, maintaining cell viability.

During testicular development and spermatogenesis progression, there is constant cell renewal. Testicular apoptosis is crucial to control the number and eliminate defective germ cells during this process, and therefore, damage to cellular DNA can be observed. Under *in vitro* conditions, there may be insufficient diffusion of nutrients and/or deficient gas exchange that can lead to apoptosis by stimulating the production of reactive oxygen species (Ibtisham *et al.* 2021). In this study, we found that there was an increase in the percentage of testicular cells with damaged DNA throughout the culture period. However, the ORG group without the addition of FGF maintained DNA integrity and a low percentage of damaged cells in all periods analyzed. In prepubertal pigs, Ibtisham *et al.* (2023), found that the addition of FGF to the culture medium promoted a higher percentage of TUNEL-positive cells, indicative of damaged DNA/apoptosis, when compared to samples cultured without growth factors or supplemented with GDNF. The use of GDNF in the IVC medium of testicular fragments of prepubertal collared peccaries has already been established and is considered essential for maintaining viable conditions for testicular adaptation (Silva *et al.* 2024).

Ki-67 is a nuclear protein that, in humans, is encoded by the MKI67 gene and is closely related to cell proliferation (Scholzen & Gerdes 2000). Our results of Ki-67-positive cells showed that in the first 14 days, the ORG without addition of FGF was similar to the uncultured control group, and the other treatments showed a reduction in the number of positive cells. At 28 days, it was observed that the addition of FGF to ORG increased cell proliferation, becoming similar to the uncultured control, and that the 3D system was able to increase cell proliferation, independent of the addition of FGF. In cultured testicular cells from domestic pigs, FGF had a negative effect on the expression of pluripotency and gonocyte marker genes (Kuijk *et al.* 2009). The authors believe that FGF may impair germ cell self-renewal in primary testicular cell cultures and indicate that male germ cell proliferation may not be supported by the presence of FGF (Kuijk *et al.* 2009). In Leydig cells, Duan *et al.* (2019) suggest that FGF stimulates the proliferation of Leydig stem and progenitor cells but blocks their differentiation. Thus, FGF appears to play an important role in these cells before differentiation but may be detrimental after this period.

In addition to organotypic and three-dimensional culture systems, microfluidic platforms represent a promising alternative for *in vitro* spermatogenesis and have been reported by several authors. Komeya *et al.* (2016) developed a microfluidic device that maintained mouse testis tissue for over 6 months, successfully producing fertile spermatozoa capable of fertilization. Later, a pumpless hydrostatic version of the system achieved similar results while simplifying device operation (Komeya *et al.* 2017). AbuMadighem *et al.* (2022) designed a 3D testis-on-a-chip, supporting meiotic progression to haploid cells using methylcellulose scaffolds over a 7-week culture period. Sharma *et al.* (2022*a*,*b*) extended this approach to non-human primates and human testicular tissue, demonstrating short-term viability and hormonal responsiveness in culture for up to 11 days. Given these advances, a logical next step would be the long-term evaluation of such microfluidic systems, particularly for supporting complete spermatogenesis in relevant animal models.

Overall, the ORG system without FGF addition efficiently provided conditions for maintaining the histomorphology, viability, and DNA integrity of peccary testicular fragments during IVC. However, the 3D system, regardless of the presence of FGF, impaired the DNA integrity of testicular cells in all periods analyzed. Furthermore, we highlight the advantage of ORG over 3D, as it requires less time for preparation, does not require the use of culture medium in the dilution of agarose, and facilitates the manipulation of fragments. Thus, we found promising results regarding the ideal culture conditions for testicular tissue fragments of prepubertal collared peccaries and the interaction of FGF with other components already established for this species.

In conclusion, the organotypic system appears to be ideal for the long-term culture of testicular fragments of prepubertal collared peccaries. Moreover, the FGF supplementation seems not to be essential for peccary testicular tissue IVC. This information fills gaps in the understanding of the factors that regulate *in vitro* spermatogenesis in this species, and the future production of sperm *in vitro*.

## Declaration of interest

The authors declare that they have no conflicts of interest. P Comizzoli is an associate editor of *Reproduction & Fertility* and was not involved in the review or editorial process for this paper, on which he is listed as an author.

## Funding

This study was financed in part by the Coordenação de Aperfeiçoamento de Pessoal de Nível Superior – Brazil (CAPES, Finance Code 001), and the National Counsel of Technological and Scientific Development CNPq proccess number: (CNPq, Process no. 306409/2022-4). MF Oliveira, AF Pereira, and AR Silva are CNPq investigators.

## Author contribution statement

AGP, AMS, LGPB, JLCA, and GSCB contributed to the conceptualization and methodology of the study. AFP, MFO, CEBM, PC, and ARS were involved in the investigation and resource management. AGP and AMS contributed to data analysis. AGP was responsible for writing the original draft of the manuscript. FK, ZN, MB, and HG reviewed and edited the manuscript. ARS managed the project administration. All authors have read and approved the final manuscript.
